# Functional Role of Gonadotrope Plasticity and Network Organization

**DOI:** 10.3389/fendo.2017.00223

**Published:** 2017-09-07

**Authors:** Brian S. Edwards, Colin M. Clay, Buffy S. Ellsworth, Amy M. Navratil

**Affiliations:** ^1^Department of Zoology and Physiology, University of Wyoming, Laramie, WY, United States; ^2^Department of Biomedical Science, Colorado State University, Fort Collins, CO, United States; ^3^Department of Physiology, Southern Illinois University Carbondale, Carbondale, IL, United States

**Keywords:** gonadotropin-releasing hormone receptor, actin cytoskeleton, extracellular signal regulated kinase signaling, gonadotrope cell signaling, luteinzing hormone, network dynamics

## Abstract

Gonadotrope cells of the anterior pituitary are characterized by their ability to mount a cyclical pattern of gonadotropin secretion to regulate gonadal function and fertility. Recent *in vitro* and *in vivo* evidence suggests that gonadotropes exhibit dramatic remodeling of the actin cytoskeleton following gonadotropin-releasing hormone (GnRH) exposure. GnRH engagement of actin is critical for gonadotrope function on multiple levels. First, GnRH-induced cell movements lead to spatial repositioning of the *in vivo* gonadotrope network toward vascular endothelium, presumably to access the bloodstream for effective hormone release. Interestingly, these plasticity changes can be modified depending on the physiological status of the organism. Additionally, GnRH-induced actin assembly appears to be fundamental to gonadotrope signaling at the level of extracellular signal-regulated kinase (ERK) activation, which is a well-known regulator of luteinizing hormone (LH) β-subunit synthesis. Last, GnRH-induced cell membrane projections are capable of concentrating LHβ-containing vesicles and disruption of the actin cytoskeleton reduces LH secretion. Taken together, gonadotrope network positioning and LH synthesis and secretion are linked to GnRH engagement of the actin cytoskeleton. In this review, we will cover the dynamics and organization of the *in vivo* gonadotrope cell network and the mechanisms of GnRH-induced actin-remodeling events important in ERK activation and subsequently hormone secretion.

## Introduction

Gonadotrope cells are a population of endocrine cells located in the anterior pituitary that are responsible for regulating the reproductive axis (1, 2). Gonadotropin-releasing hormone (GnRH) is synthesized in hypothalamic neurons and secreted in a pulsatile manner toward the fenestrated capillaries in the median eminence. Following release, GnRH is transported *via* the hypophysial portal vessels to the anterior pituitary where it binds to the GnRH receptor (GnRHR) located on gonadotrope cells. Stimulation of the GnRHR culminates in the synthesis and secretion of four main gene products: the common glycoprotein α-subunit, the hormone-specific luteinizing hormone (LH) β subunit, follicle-stimulating hormone (FSH) β-subunit, and the GnRHR (1–3). The heterodimeric glycoproteins, LH and FSH, are then released into systemic circulation where they regulate gonadal development and function by stimulating steroidogenesis, gametogenesis, folliculogenesis, and ovulation (4, 5).

Depending on the phenotypic markers used to identify gonadotropes, the population undergoes dynamic changes in both size and numbers depending on the stage of the estrous cycle (6–8). For example, gonadotropes are thought to represent approximately 5–7% of total anterior pituitary cells during diestrus but can increase upwards to 15% in proestrus (8, 9). Additionally, evidence suggests that gonadotropes are a heterogeneous population of cells that can be classified as small, medium, and large (10–12). Gonadotropes that are large are bihormonal and enriched during estrus (13). Gonadotrope cells are also organized in homotypic and heterotypic cellular networks that can adapt to changing physiological conditions to generate coordinated hormone pulsatility (14–16). Examples of adaptable mechanisms in gonadotropes include cell morphology, migration, and positioning to vasculature; all of which requires a dynamic actin cytoskeleton.

The actin cytoskeleton plays an important role in cell division, motility, and intracellular trafficking of vesicles. The actin cytoskeleton has been extensively studied in the nervous system where it is important in synaptic morphology, function, vesicle mobilization, and recycling (17–20). Similarly, in secretory cells such as gonadotropes, an intact actin cytoskeleton is important in the regulated release of vesicular hormones and the replenishment of these vesicles with reserve vesicles (21–23). Thus, gonadotrope network organization and plasticity is essential to the optimization of proper reproductive function. In this review, we will highlight gonadotrope population networks and organization, GnRH-mediated actin reorganization events, and functionally linking these events with mitogen-activated protein kinase (MAPK) activation and subsequent gonadotropin secretion.

## Gonadotrope Development and Organization

The anterior pituitary is a complex endocrine gland that secretes multiple hormones to control homeostasis, growth, lactation, and reproduction. It is composed of five distinct endocrine cell types: gonadotropes, thyrotropes, corticotropes, somatotropes, and lactotropes (24). During murine development, organogenesis of the pituitary commences at embryonic day (e) 9.0 with a focal dorsal invagination of somatic oral ectoderm (Rathke’s pouch) to form the anterior and intermediate lobes (25). Lineage commitment and differentiation of pituitary cells initiates at e12.5 in a sequential manner and are orchestrated by combinatorial expression of cell type-specific transcription factors, epigenetic modifications, and cell–cell interactions (24, 26, 27). Gonadotrope cells are the last of the anterior pituitary cell lineages to undergo terminal differentiation with expression of the *Lhb* transcript occurring on e16.5, then *Fshb* on e17.5. Gonadotropes begin to become clustered and are localized to the central mediolateral region by e18.5 (14, 24, 27).

During development, it has been suggested that organization of latter differentiating anterior pituitary endocrine cell types (i.e., gonadotropes) are directed by earlier developing endocrine cell types (14). Indeed, corticotropes, which have been detected in mice at e13.5, are thought to direct the differentiation and clustering of gonadotropes (14, 15). The organization of the heterotypic network between gonadotropes and corticotropes occurs along the ventral surface of the anterior pituitary and is thought that these cells maintain direct contact throughout adulthood. In contrast, the homotypic network of gonadotropes develops along the dorsal surface of the anterior pituitary with little contact with other endocrine cell types (14). Interestingly, pituitaries that are deficient in corticotropes, *Tpit^−/−^* pituitaries display a decrease in gonadotrope cell volume and an increase in gonadotrope number due to an alternate cell fate adopted by their common precursor (28). A role for inter-connected networks was also highlighted between lactotropes and gonadotropes where ablation of gonadotropes resulted in modifications of lactotrope development and organization (29). Thus, network inter-connectivity between endocrine cell types may act as a scaffold that serves to organize and establish gonadotrope networks.

Postnatally, gonadotrope populations have been shown to be homogenously distributed throughout (lateral, caudal, rostral) the anterior pituitary when imaging whole-mount preparations of entire pituitary glands from prepubertal mice (30). However, following reproductive maturation, there is an increased density of gonadotropes in the rostral region relative to the lateral and ventral regions of the anterior pituitary. Furthermore, postpubertal gonadotrope populations have been characterized as being organized in string like clusters on both the ventral and dorsal surfaces of the anterior pituitary (14). Thus, plasticity within the gonadotrope population may be key for mounting appropriate responses to fluctuating hormone levels that occur as mice transition from pre- to postpuberty. Toward this end, priming gonadotropes with long-term estradiol treatment increased cellular plasticity and responsiveness to GnRH (30). Interestingly, the population of gonadotropes as a whole in sexually mature mice also display a high degree of plasticity depending on the physiological demands. This is demonstrated in lactating mice where gonadotropes reside in clusters in the lateral and ventral areas and not in the rostral region (30). Taken together, it is clear that gonadotrope networks exhibit a continuous plasticity that is pertinent to producing a proper response to changing physiological conditions.

## Gonadotrope Plasticity *In Vivo*

A primary goal of cellular secretory elements of endocrine glands is directed secretion of hormone into the blood stream. As such, endocrine cells are often embedded in connective tissue surrounded by rich vascular networks. In particular, it has long been evident that gonadotrope cells display considerable surface area in close apposition to capillary endothelium (14, 30). Such an arrangement presumably allows for efficient and robust delivery of gonadotropin into the circulation. It is reasonable to predict that gonadotrope “priming” reflects multiple events that include enhanced GnRH responsiveness, mobilization of secretory granules and, perhaps, increased apposition of the basolateral secretory surface area of gonadotropes. Collectively then, each of these would contribute to placing both gonadotropes and secretory granules in the most effective position for maximal release of hormone in response to GnRH stimulation. According to this paradigm, GnRH not only elicits exocytosis of secretory granules from gonadotropes but also contributes to organizing these cells in the most favorable spatial orientation to achieve the rapid and pronounced increase in circulating gonadotropin concentrations.

Gonadotropes are characterized by their ability to mount a cyclical pattern of hormone secretion, an event critical in the production of the preovulatory LH surge in females (2, 3, 31, 32). Previous evidence suggests that these cells display both structural and functional plasticity throughout the female reproductive cycle (6, 9, 30). Under conditions of GnRH and, perhaps, steroid stimulation, morphological rearrangements of gonadotropes are elicited leading to the development of cellular processes or projections that extend toward capillary sinusoids. As early as 1985, Dr. Gwen Childs noted that GnRH stimulated gonadotropes developed processes during peak LH secretory episodes (7). Osamura and colleagues in Japan also demonstrated a similar phenomenon based on three dimensional reconstructions of pituitary vasculature and endocrine cells (33, 34). Previous live cell studies of *ex vivo* pituitary slices have shown that gonadotropes display a high degree of plasticity in the face of neuroendocrine stimulation (30, 35). GnRH exposure to murine pituitary slices leads cell processes and spatial repositioning of GFP-labeled gonadotropes using the *ex vivo* paradigm (35). The stimulation-dependent plasticity displayed by gonadotropes is thought to lead to increased association between gonadotropes and the microvasculature of the pituitary (30). Spatial positioning of gonadotropes reveals a much closer proximity to vasculature when compared to corticotropes (14), and there is evidence that gonadotropes can have a close spatial association with more than one blood vessel through multiple cellular projections. It should be noted that the GnRH-induced cellular projections extending toward blood vessels contain LH secretory granules, which may increase the secretory impact of gonadotropes (36).

Gonadotropin-releasing hormone-induced plasticity in gonadotropes creates transient cellular structures in the form of lamellipodia, membrane ruffles, and filopodia. The actin cytoskeleton supports these membrane remodeling events by assembling actin monomers to form filamentous actin (17, 37, 38). We have previously found that GnRH induces rapid dynamic engagement of the actin cytoskeleton within 1 min of treatment (35). However, pretreatment with a pharmacological disruptor of the actin cytoskeleton, jasplakinolide (Jas), blunts GnRH-induced membrane remodeling events (35). Not only does the actin cytoskeleton play an important role in structural support and cell migration but it is also important for coordinating the trafficking and release of secretory vesicles in endocrine cells. We have previously shown that upon GnRH stimulation of primary murine gonadotrope cells, there is an approximate 3.5-fold increase in LH secretion (36). In contrast, GnRH stimulation to primary murine pituitary cells that are pretreated with Jas results in a significant reduction in LH secretion with no difference compared to vehicle (36). Thus, the GnRH-mediated plasticity is critical in maintaining physiological levels of LH and to spatially align responsive gonadotropes in close proximity to the pituitary vasculature for secretory events.

Gonadotrope plasticity is also pertinent in establishing an organized network throughout the anterior pituitary. Network organization is a critical aspect in the maintenance of reproduction as gonadotropes must orchestrate hormone secretory events in the face of changing physiological demands. In order for proper gonadotrope organization, it is thought that communication and interaction between endocrine and non-endocrine networks is an underlying mechanism. Specifically, folliculostellate cells predominantly communicate through gap junctions and paracrine and autocrine signaling with endocrine cells in the anterior pituitary (15, 39). Additionally, the number of gap junctions between folliculostellate cells and the altered morphological relationship with hormonal cells in the anterior pituitary also provides additional evidence of functional plasticity in this non-hormonal cell type (40–42). Overall, the large-scale gonadotrope reorganization and interaction with non-hormonal cells may be the key in mounting a proper response to changing physiological conditions through connections with one or more blood vessels *via* their protrusions.

## Gonadotrope Signaling to Actin

Gonadotropin-releasing hormone actions are modulated through the GnRH receptor (GnRHR), a G-protein-coupled receptor found on the plasma membrane of gonadotropes. Upon activation, the GnRHR undergoes a conformational change that promotes the activation of the heterotrimeric G-proteins, specifically, Gα_q/11_. Activation of Gα_q/11_ activates phospholipase Cβ1, which hydrolyzes phosphatidylinositol-4,5-bisphosphate (PIP_2_) to generate inositol-1,4,5-triphosphate (IP_3_) and diacylglycerol (DAG). IP_3_ interaction with the IP_3_ receptor induces an elevation of intracellular Ca^2+^ from the endoplasmic reticulum, while DAG activates one or more isoforms of PKC (43, 44) that initiate Ca^2+^ influx through activation of voltage-gated L-type Ca^2+^ channels (VGCCs) (45–47). These upstream events underlie GnRH activation of extracellular signal-regulated kinase (ERK), the MAPK predominantly involved in regulating LHβ synthesis (48–50).

The actin cytoskeleton is a dense meshwork of protein polymers that undergoes cycles of assembly and disassembly and is regulated by a number of actin-associated proteins (37). Cortactin is a filamentous actin-binding protein that acts as an actin-scaffolding protein to mediate actin polymerization (51, 52). Cortactin mediates actin polymerization by binding actin-related protein (Arp) 2/3 complex, a nucleating factor that serves to facilitate actin filament branching, through a three amino acid motif in its amino terminus (52). Furthermore, cortactin is a target of multiple tyrosine and serine/threonine kinases (53, 54). Our laboratory has previously shown that cortactin activation is required for GnRH-induced plasticity in αT3-1 gonadotropes, and that src-induced tyrosine phosphorylation of cortactin is key in facilitating association of Arp3 to effectively engage the actin cytoskeleton (36).

In addition to regulating actin polymerization, cortactin may also serve as a functional link between intracellular signaling cascades and actin assembly events (53–55). Interestingly, disrupting the actin cytoskeleton with Jas resulted in a loss of GnRH-induced ERK phosphorylation. However, GnRH-induced cell movement and projections is not inhibited by the MAPK kinase 1 inhibitor PD98059. Collectively suggesting that in αT3-1 cells, ERK activation is not a prerequisite for actin reorganization, but an intact actin cytoskeleton is required in the activation of ERK (35, 36). Consistent with this work, HEK293 cells expressing the GnRHR showed altered cellular morphology and cytoskeletal reorganization following treatment with GnRH. In addition, activation of ERK was significantly reduced following cytoskeletal disruption (56). The precise mechanism of how actin engagement impacts ERK activation remains unclear although data suggests that PKC is working downstream of the actin cytoskeleton. In support of this, direct activation of PKC with phorbol 12-myristate 13-acetate was not sufficient to induce cytoskeletal remodeling suggesting that PKC is working downstream of the actin cytoskeleton to facilitate activation of ERK in gonadotropes. Recent work also supports the notion that actin reorganization may be important for GnRH-mediated opening of L-type calcium channels (47)—the key calcium signal leading to ERK activation (46, 47).

Dynamin, a large GTPase and proline-rich domain-containing protein, possesses mechanochemical properties important in membrane remodeling events and fission (57). Many of these functions of dynamin appears to be associated with remodeling of the actin cytoskeleton (58); however, the mechanism by which it does so remains unclear. Overexpression of dominant-negative dynamin mutant proteins impaired in hydrolyzing GTP (K44A) perturbs many F-actin-rich cellular structures (59–61). Consistent with this data, αT3-1 cells transfected with K44A resulted in a loss of GnRH-induced actin remodeling events (62). Our group also demonstrated that pharmacological inhibition of dynamin GTPase activity, using both dynasore and dyngo, not only perturbed GnRH-induced actin reorganization but also significantly suppressed ERK activation (63). Thus, highlighting the importance of dynamin GTPase activity in actin reorganization and subsequent MAPK activation. In addition, the actin-binding protein, cortactin, not only enhances dynamin GTPase activity but also binds dynamin through its C-terminal SH3-domain (64). It is also well known that dynamin and cortactin colocalize in podosomes (65), membrane ruffles (66), and actin comets (67). Similarly, our group highlighted that upon GnRH stimulation, cortactin and dynamin are redistributed and become colocalized in areas indicative of high actin reorganization in αT3-1 cells (63). In addition to regulating Tyr phosphorylation of cortactin, there is also evidence that src induces Tyr phosphorylation of dynamin (62, 68). Thus, GnRH-induced gonadotrope plasticity may be modulated through the interaction of dynamin and cortactin to effectively engage the actin cytoskeleton to subsequently regulate PKC activation, VGCC opening, and ERK phosphorylation (47, 63). Clearly, the functionality and mechanism by which dynamin regulates gonadotrope plasticity warrants further investigation.

Although our group and others have started to unravel the signaling intermediates, GnRH utilizes to engage the actin cytoskeleton, identification of the full cohort of intermediates remains unclear. Recent work suggests that mammalian target of rapamycin (mTOR) also signals to the actin cytoskeleton to regulate cellular morphology both *in vitro* and *in vivo* (69, 70). mTOR is a serine/threonine protein kinase that forms two distinct complexes, mTORC1 and mTORC2. Our recent work using the LβT2 gonadotrope cell line establishes a specific role for mTORC2 in regulating membrane remodeling events (71). Pharmacological inhibition of mTORC2-blunted GnRH-mediated actin reorganization and similarly attenuated activation of ERK and LHβ gene expression (71). Although we have established an additional key intermediate linking GnRHR signaling to actin remodeling and ERK activation, the upstream signaling molecules regulating activation of mTORC2 in LβT2 cells remains unknown. It has been previously demonstrated that the Rho GTPase, Rac1, binds to and activates mTORC2 and also facilitates localization to the plasma membrane (72). GnRH also modulates LβT2 cell morphology and migration through Rho family members (73). Thus, Rac1 is likely a strong candidate involved in mediating mTORC2 activation and subsequent engagement of the actin cytoskeleton in gonadotrope cells. Taken together, GnRH-mediated actin cytoskeletal reorganization is controlled by multiple signaling networks to insure proper reproductive functioning.

## Conclusion

The gonadotrope population displays profound plasticity that is present during late stages of embryological development and continues into adulthood (14, 15). The plasticity in the population is not only dependent on fluctuating hormone levels and reproductive status but also other endocrine cellular networks acting as a guidance scaffold. However, the gonadotrope plasticity in an individual cell is dependent on an intact dynamic actin cytoskeleton that is directed by multiple signaling intermediates. The actin cytoskeleton in gonadotropes serves a critical function in maintaining competence of the hypothalamic–pituitary–gonadal axis and mammalian fertility. We highlighted that GnRH engages the actin cytoskeleton to not only increase cell movement but also causes membrane remodeling events in the form of membrane ruffles, filopodia, and lamellipodia to potentially gain increased access to the pituitary vasculature (30). We suggest that cortactin and dynamin form an actin remodeling protein complex that functionally links neuroendocrine stimulation and actin polymerization (35, 36, 63). We also underscore mTORC2 as an additional signaling intermediate important in regulating membrane remodeling events and subsequent MAPK activation in gonadotropes (71). However, despite our data emphasizing gonadotrope plasticity and associated proteins; the mechanisms by which actin polymerization results in activation of ERK upstream of PKC remains largely undefined.

## Author Contributions

All authors have contributed to the writing of this review, and all have read and approved the final manuscript.

## Conflict of Interest Statement

The authors declare that the research was conducted in the absence of any commercial or financial relationships that could be construed as a potential conflict of interest.

## Abbreviations

GnRH, gonadotropin-releasing hormone; GnRHR, gonadotropin-releasing hormone receptor; LH, luteinizing hormone; FSH, follicle-stimulating hormone; MAPK, mitogen-activated protein kinase; ERK, extracellular signal regulated kinase; Arp 2/3, actin-related protein 2 and 3; mTORC2, mammalian target of rapamycin complex 2.

## References

[1] Desjardins C Endocrine signaling and male reproduction. Biol Reprod (1981) 24:1–21. 10.1095/biolreprod24.1.1 6781545

[2] Brinkley HJ Endocrine signaling and female reproduction. Biol Reprod (1981) 24:22–43. 10.1095/biolreprod24.1.22 6781548

[3] Clayton RN Catt KJ Gonadotropin-releasing hormone receptors: characterization, physiological regulation, and relationship to reproductive function. Endocr Rev (1981) 2:186–209. 10.1210/edrv-2-2-186 6271547

[4] Counis R Laverriere JN Garrel G Bleux C Cohen-Tannoudji J Lerrant Y Gonadotropin-releasing hormone and the control of gonadotrope function. Reprod Nutr Dev (2005) 45:243–54. 10.1051/rnd:2005017 15982451

[5] Gharib SD Wierman ME Shupnik MA Chin WW Molecular biology of the pituitary gonadotropins. Endocr Rev (1990) 11:177–99. 10.1210/edrv-11-1-177 2108012

[6] Childs GV Unabia G Lloyd J. Recruitment and maturation of small subsets of luteinizing hormone gonadotropes during the estrous cycle. Endocrinology (1992) 130:335–44. 10.1210/endo.130.1.1727707 1727707

[7] Childs GV. Shifts in gonadotropin storage in cultured gonadotropes following GnRH stimulation, in vitro. Peptides (1985) 6:103–7. 10.1016/0196-9781(85)90084-1 3921943

[8] Smith PF Frawley LS Neill JD. Detection of LH release from individual pituitary cells by the reverse hemolytic plaque assay: estrogen increases the fraction of gonadotropes responding to GnRH. Endocrinology (1984) 115:2484–6. 10.1210/endo-115-6-2484 6389096

[9] Childs GV Unabia G Lee BL Rougeau D. Heightened secretion by small and medium-sized luteinizing hormone (LH) gonadotropes late in the cycle suggests contributions to the LH surge or possible paracrine interactions. Endocrinology (1992) 130:345–52. 10.1210/endo.130.1.1727708 1727708

[10] Childs G Ellison D Foster L Ramaley JA. Postnatal maturation of gonadotropes in the male rat pituitary. Endocrinology (1981) 109:1683–92. 10.1210/endo-109-5-1683 6271539

[11] Childs GV Unabia G Tibolt R Lloyd JM Cytological factors that support nonparallel secretion of luteinizing hormone and follicle-stimulating hormone during the estrous cycle. Endocrinology (1987) 121:1801–13. 10.1210/endo-121-5-1801 2444429

[12] Childs GV Hyde C Naor Z Catt K. Heterogeneous luteinizing hormone and follicle-stimulating hormone storage patterns in subtypes of gonadotropes separated by centrifugal elutriation. Endocrinology (1983) 113:2120–8. 10.1210/endo-113-6-2120 6196182

[13] Lloyd JM Childs GV. Differential storage and release of luteinizing hormone and follicle-releasing hormone from individual gonadotropes separated by centrifugal elutriation. Endocrinology (1988) 122:1282–90. 10.1210/endo-122-4-1282 3126034

[14] Budry L Lafont C El Yandouzi T Chauvet N Conejero G Drouin J Related pituitary cell lineages develop into interdigitated 3D cell networks. Proc Natl Acad Sci U S A (2011) 108:12515–20. 10.1073/pnas.1105929108 21746936 PMC3145718

[15] Le Tissier PR Hodson DJ Lafont C Fontanaud P Schaeffer M Mollard P. Anterior pituitary cell networks. Front Neuroendocrinol (2012) 33:252–66. 10.1016/j.yfrne.2012.08.002 22981652

[16] Schaeffer M Hodson DJ Lafont C Mollard P. Endocrine cells and blood vessels work in tandem to generate hormone pulses. J Mol Endocrinol (2011) 47:R59–66. 10.1530/JME-11-0035 21622530

[17] Doussau F Augustine GJ. The actin cytoskeleton and neurotransmitter release: an overview. Biochimie (2000) 82:353–63. 10.1016/S0300-9084(00)00217-0 10865123

[18] Sankaranarayanan S Atluri PP Ryan TA. Actin has a molecular scaffolding, not propulsive, role in presynaptic function. Nat Neurosci (2003) 6:127–35. 10.1038/nn1002 12536209

[19] Prekeris R Terrian DM. Brain myosin V is a synaptic vesicle-associated motor protein: evidence for a Ca2+-dependent interaction with the synaptobrevin-synaptophysin complex. J Cell Biol (1997) 137:1589–601. 10.1083/jcb.137.7.1589 9199173 PMC2137828

[20] Evans LL Lee AJ Bridgman PC Mooseker MS. Vesicle-associated brain myosin-V can be activated to catalyze actin-based transport. J Cell Sci (1998) 111(Pt 14):2055–66. 9645952 10.1242/jcs.111.14.2055

[21] Adams TE Nett TM Interaction of GnRH with anterior pituitary. III. Role of divalent cations, microtubules and microfilaments in the GnRH activated gonadotroph. Biol Reprod (1979) 21:1073–86. 10.1095/biolreprod21.5.1073 229920

[22] Stojilkovic SS Chang JP Izumi S Tasaka K Catt KJ. Mechanisms of secretory responses to gonadotropin-releasing hormone and phorbol esters in cultured pituitary cells. Participation of protein kinase C and extracellular calcium mobilization. J Biol Chem (1988) 263:17301–6. 2460461

[23] Izumi T Kasai K Gomi H. Secretory vesicle docking to the plasma membrane: molecular mechanism and functional significance. Diabetes Obes Metab (2007) 9(Suppl 2):109–17. 10.1111/j.1463-1326.2007.00789.x 17919185

[24] Zhu X Gleiberman AS Rosenfeld MG. Molecular physiology of pituitary development: signaling and transcriptional networks. Physiol Rev (2007) 87:933–63. 10.1152/physrev.00006.2006 17615393

[25] Kelberman D Rizzoti K Lovell-Badge R Robinson IC Dattani MT. Genetic regulation of pituitary gland development in human and mouse. Endocr Rev (2009) 30:790–829. 10.1210/er.2009-0008 19837867 PMC2806371

[26] Zhu X Wang J Ju BG Rosenfeld MG. Signaling and epigenetic regulation of pituitary development. Curr Opin Cell Biol (2007) 19:605–11. 10.1016/j.ceb.2007.09.011 17988851 PMC2796608

[27] Davis SW Ellsworth BS Perez Millan MI Gergics P Schade V Foyouzi N Pituitary gland development and disease: from stem cell to hormone production. Curr Top Dev Biol (2013) 106:1–47. 10.1016/B978-0-12-416021-7.00001-8 24290346 PMC4039019

[28] Pulichino AM Vallette-Kasic S Tsai JP Couture C Gauthier Y Drouin J. Tpit determines alternate fates during pituitary cell differentiation. Genes Dev (2003) 17:738–47. 10.1101/gad.1065703 12651892 PMC196016

[29] Seuntjens E Vankelecom H Quaegebeur A Vande Vijver V Denef C. Targeted ablation of gonadotrophs in transgenic mice affects embryonic development of lactotrophs. Mol Cell Endocrinol (1999) 150:129–39. 10.1016/S0303-7207(99)00011-8 10411307

[30] Alim Z Hartshorn C Mai O Stitt I Clay C Tobet S Gonadotrope plasticity at cellular and population levels. Endocrinology (2012) 153:4729–39. 10.1210/en.2012-1360 22893721 PMC3685717

[31] Bliss SP Navratil AM Xie J Roberson MS. GnRH signaling, the gonadotrope and endocrine control of fertility. Front Neuroendocrinol (2010) 31:322–40. 10.1016/j.yfrne.2010.04.002 20451543 PMC2923852

[32] Knobil E On the control of gonadotropin secretion in the rhesus monkey. Recent Prog Horm Res (1974) 30:1–46. 4210416 10.1016/b978-0-12-571130-2.50005-5

[33] Itoh J Serizawa A Kawai K Ishii Y Teramoto A Osamura RY. Vascular networks and endothelial cells in the rat experimental pituitary glands and in the human pituitary adenomas. Microsc Res Tech (2003) 60:231–5. 10.1002/jemt.10261 12539177

[34] Itoh J Kawai K Serizawa A Yasumura K Ogawa K Osamura RY A new approach to three-dimensional reconstructed imaging of hormone-secreting cells and their microvessel environments in rat pituitary glands by confocal laser scanning microscopy. J Histochem Cytochem (2000) 48:569–78. 10.1177/002215540004800414 10727298

[35] Navratil AM Knoll JG Whitesell JD Tobet SA Clay CM. Neuroendocrine plasticity in the anterior pituitary: gonadotropin-releasing hormone-mediated movement in vitro and in vivo. Endocrinology (2007) 148:1736–44. 10.1210/en.2006-1153 17218416

[36] Navratil AM Dozier MG Whitesell JD Clay CM Roberson MS. Role of cortactin in dynamic actin remodeling events in gonadotrope cells. Endocrinology (2014) 155:548–57. 10.1210/en.2012-1924 24274984 PMC3891938

[37] Pollard TD Cooper JA. Actin, a central player in cell shape and movement. Science (2009) 326:1208–12. 10.1126/science.1175862 19965462 PMC3677050

[38] Porat-Shliom N Milberg O Masedunskas A Weigert R Multiple roles for the actin cytoskeleton during regulated exocytosis. Cell Mol Life Sci (2013) 70:2099–121. 10.1007/s00018-012-1156-5 22986507 PMC4052552

[39] Denef C. Paracrinicity: the story of 30 years of cellular pituitary crosstalk. J Neuroendocrinol (2008) 20:1–70. 10.1111/j.1365-2826.2007.01616.x 18081553 PMC2229370

[40] Shirasawa N Kihara H Yamaguchi S Yoshimura F. Pituitary folliculo-stellate cells immunostained with S-100 protein antiserum in postnatal, castrated and thyroidectomized rats. Cell Tissue Res (1983) 231:235–49. 10.1007/BF00222177 6850801

[41] Kurono C Intercellular communication within the rat anterior pituitary gland: VI. Development of gap junctions between folliculo-stellate cells under the influence of ovariectomy and sex steroids in the female rat. Anat Rec (1996) 244:366–73. 10.1002/(SICI)1097-0185(199603)244:3<366::AID-AR8>3.3.CO;2-R 8742701

[42] Soji T Nishizono H Yashiro T Herbert DC. Intercellular communication within the rat anterior pituitary gland. III. Postnatal development and periodic changes of cell-to-cell communications in female rats. Anat Rec (1991) 231:351–7. 10.1002/ar.1092310309 1763817

[43] Kaiser UB Conn PM Chin WW Studies of gonadotropin-releasing hormone (GnRH) action using GnRH receptor-expressing pituitary cell lines. Endocr Rev (1997) 18:46–70. 10.1210/er.18.1.46 9034786

[44] Stojilkovic SS Reinhart J Catt KJ Gonadotropin-releasing hormone receptors: structure and signal transduction pathways. Endocr Rev (1994) 15:462–99. 10.1210/edrv-15-4-462 7988482

[45] Mulvaney JM Roberson MS. Divergent signaling pathways requiring discrete calcium signals mediate concurrent activation of two mitogen-activated protein kinases by gonadotropin-releasing hormone. J Biol Chem (2000) 275:14182–9. 10.1074/jbc.275.19.14182 10799494

[46] Mulvaney JM Zhang T Fewtrell C Roberson MS. Calcium influx through L-type channels is required for selective activation of extracellular signal-regulated kinase by gonadotropin-releasing hormone. J Biol Chem (1999) 274:29796–804. 10.1074/jbc.274.42.29796 10514457

[47] Dang AK Murtazina DA Magee C Navratil AM Clay CM Amberg GC. GnRH evokes localized subplasmalemmal calcium signaling in gonadotropes. Mol Endocrinol (2014) 28:2049–59. 10.1210/me.2014-1208 25333516 PMC4250365

[48] Roberson MS Misra-Press A Laurance ME Stork PJ Maurer RA. A role for mitogen-activated protein kinase in mediating activation of the glycoprotein hormone alpha-subunit promoter by gonadotropin-releasing hormone. Mol Cell Biol (1995) 15:3531–9. 10.1128/MCB.15.7.3531 7791760 PMC230590

[49] Weck J Fallest PC Pitt LK Shupnik MA. Differential gonadotropin-releasing hormone stimulation of rat luteinizing hormone subunit gene transcription by calcium influx and mitogen-activated protein kinase-signaling pathways. Mol Endocrinol (1998) 12:451–7. 10.1210/mend.12.3.0070 9514161

[50] White BR Duval DL Mulvaney JM Roberson MS Clay CM. Homologous regulation of the gonadotropin-releasing hormone receptor gene is partially mediated by protein kinase C activation of an activator protein-1 element. Mol Endocrinol (1999) 13:566–77. 10.1210/mend.13.4.0262 10194763

[51] Weaver AM Karginov AV Kinley AW Weed SA Li Y Parsons JT Cortactin promotes and stabilizes Arp2/3-induced actin filament network formation. Curr Biol (2001) 11:370–4. 10.1016/S0960-9822(01)00098-7 11267876

[52] Uruno T Liu J Zhang P Fan Y Egile C Li R Activation of Arp2/3 complex-mediated actin polymerization by cortactin. Nat Cell Biol (2001) 3:259–66. 10.1038/35060051 11231575

[53] Ammer AG Weed SA. Cortactin branches out: roles in regulating protrusive actin dynamics. Cell Motil Cytoskeleton (2008) 65:687–707. 10.1002/cm.20296 18615630 PMC2561250

[54] Cosen-Binker LI Kapus A. Cortactin: the gray eminence of the cytoskeleton. Physiology (Bethesda) (2006) 21:352–61. 10.1152/physiol.00012.2006 16990456

[55] Weed SA Parsons JT. Cortactin: coupling membrane dynamics to cortical actin assembly. Oncogene (2001) 20:6418–34. 10.1038/sj.onc.1204783 11607842

[56] Davidson L Pawson AJ Millar RP Maudsley S. Cytoskeletal reorganization dependence of signaling by the gonadotropin-releasing hormone receptor. J Biol Chem (2004) 279:1980–93. 10.1074/jbc.M309827200 14559894

[57] Zhang P Hinshaw JE. Three-dimensional reconstruction of dynamin in the constricted state. Nat Cell Biol (2001) 3:922–6. 10.1038/ncb1001-922 11584275

[58] Orth JD McNiven MA. Dynamin at the actin-membrane interface. Curr Opin Cell Biol (2003) 15:31–9. 10.1016/S0955-0674(02)00010-8 12517701

[59] Gray NW Kruchten AE Chen J McNiven MA. A dynamin-3 spliced variant modulates the actin/cortactin-dependent morphogenesis of dendritic spines. J Cell Sci (2005) 118:1279–90. 10.1242/jcs.01711 15741233

[60] Schafer DA Weed SA Binns D Karginov AV Parsons JT Cooper JA. Dynamin2 and cortactin regulate actin assembly and filament organization. Curr Biol (2002) 12:1852–7. 10.1016/S0960-9822(02)01228-9 12419186 PMC9590655

[61] Krueger EW Orth JD Cao H McNiven MA. A dynamin-cortactin-Arp2/3 complex mediates actin reorganization in growth factor-stimulated cells. Mol Biol Cell (2003) 14:1085–96. 10.1091/mbc.E02-08-0466 12631725 PMC151581

[62] Benard O Naor Z Seger R Role of dynamin, Src, and Ras in the protein kinase C-mediated activation of ERK by gonadotropin-releasing hormone. J Biol Chem (2001) 276:4554–63. 10.1074/jbc.M006995200 11083862

[63] Edwards BS Dang AK Murtazina DA Dozier MG Whitesell JD Khan SA Dynamin is required for GnRH signaling to L-type calcium channels and activation of ERK. Endocrinology (2016) 157:831–43. 10.1210/en.2015-1575 26696122 PMC4733113

[64] Mooren OL Kotova TI Moore AJ Schafer DA. Dynamin2 GTPase and cortactin remodel actin filaments. J Biol Chem (2009) 284:23995–4005. 10.1074/jbc.M109.024398 19605363 PMC2781994

[65] Ochoa GC Slepnev VI Neff L Ringstad N Takei K Daniell L A functional link between dynamin and the actin cytoskeleton at podosomes. J Cell Biol (2000) 150:377–89. 10.1083/jcb.150.2.377 10908579 PMC2180219

[66] McNiven MA Kim L Krueger EW Orth JD Cao H Wong TW. Regulated interactions between dynamin and the actin-binding protein cortactin modulate cell shape. J Cell Biol (2000) 151:187–98. 10.1083/jcb.151.1.187 11018064 PMC2189798

[67] Orth JD Krueger EW Cao H McNiven MA. The large GTPase dynamin regulates actin comet formation and movement in living cells. Proc Natl Acad Sci U S A (2002) 99:167–72. 10.1073/pnas.012607899 11782546 PMC117533

[68] Bruzzaniti A Neff L Sanjay A Horne WC De Camilli P Baron R. Dynamin forms a Src kinase-sensitive complex with Cbl and regulates podosomes and osteoclast activity. Mol Biol Cell (2005) 16:3301–13. 10.1091/mbc.E04-12-1117 15872089 PMC1165412

[69] Jacinto E Loewith R Schmidt A Lin S Ruegg MA Hall A Mammalian TOR complex 2 controls the actin cytoskeleton and is rapamycin insensitive. Nat Cell Biol (2004) 6:1122–8. 10.1038/ncb1183 15467718

[70] Thomanetz V Angliker N Cloetta D Lustenberger RM Schweighauser M Oliveri F Ablation of the mTORC2 component rictor in brain or Purkinje cells affects size and neuron morphology. J Cell Biol (2013) 201:293–308. 10.1083/jcb.201205030 23569215 PMC3628512

[71] Edwards BS Isom WJ Navratil AM Gonadotropin releasing hormone activation of the mTORC2/Rictor complex regulates actin remodeling and ERK activity in LbetaT2 cells. Mol Cell Endocrinol (2017) 439:346–53. 10.1016/j.mce.2016.09.021 27663077 PMC5123956

[72] Saci A Cantley LC Carpenter CL. Rac1 regulates the activity of mTORC1 and mTORC2 and controls cellular size. Mol Cell (2011) 42:50–61. 10.1016/j.molcel.2011.03.017 21474067 PMC3750737

[73] Godoy J Nishimura M Webster NJ. Gonadotropin-releasing hormone induces miR-132 and miR-212 to regulate cellular morphology and migration in immortalized LbetaT2 pituitary gonadotrope cells. Mol Endocrinol (2011) 25:810–20. 10.1210/me.2010-0352 21372146 PMC3082323

